# Capillary-Based and Stokes-Based Trapping of Serial Sections for Scalable 3D-EM Connectomics

**DOI:** 10.1523/ENEURO.0328-19.2019

**Published:** 2020-04-08

**Authors:** Timothy J. Lee, Mighten C. Yip, Aditi Kumar, Colby F. Lewallen, Daniel J. Bumbarger, R. Clay Reid, Craig R. Forest

**Affiliations:** 1 Georgia Institute of Technology, G. W. Woodruff School of Mechanical Engineering, Atlanta, GA 30332; 2 Allen Institute for Brain Science, Seattle, WA 98109

**Keywords:** capillary interactions, electron microscopy, histology, hydrodynamic, serial sectioning, ultrastructure

## Abstract

Serial section electron microscopy (ssEM), a technique where volumes of tissue can be anatomically reconstructed by imaging consecutive tissue slices, has proven to be a powerful tool for the investigation of brain anatomy. Between the process of cutting the slices, or “sections,” and imaging them, however, handling 10°−10^6^ delicate sections remains a bottleneck in ssEM, especially for batches in the “mesoscale” regime, i.e., 10^2^–10^3^ sections. We present a tissue section handling device that transports and positions sections, accurately and repeatability, for automated, robotic section pick-up and placement onto an imaging substrate. The device interfaces with a conventional ultramicrotomy diamond knife, accomplishing in-line, exact-constraint trapping of sections with 100-μm repeatability. An associated mathematical model includes capillary-based and Stokes-based forces, accurately describing observed behavior and fundamentally extends the modeling of water-air interface forces. Using the device, we demonstrate and describe the limits of reliable handling of hundreds of slices onto a variety of electron and light microscopy substrates without significant defects (*n* = 8 datasets composed of 126 serial sections in an automated fashion with an average loss rate and throughput of 0.50% and 63 s/section, respectively. In total, this work represents an automated mesoscale serial sectioning system for scalable 3D-EM connectomics.

## Significance Statement

Serial section electron microscopy (ssEM), a technique where volumes of tissue can be anatomically reconstructed by imaging consecutive tissue slices, has proven to be a powerful tool for studying neuroanatomy. However, between the process of cutting the slices and imaging them, handling 10°−106 delicate slices, or “sections,” remains a bottleneck in ssEM, especially for batches in the “mesoscale” regime, i.e., 10^2^–10^3^ sections. Here, we present a section handling device that transports and positions sections for automated, robotic section pick-up and placement onto an imaging substrate. As a part of this device, we characterize a trapping technique that utilizes curvature-induced capillary-based forces and hydrodynamic Stokes drag-based forces. In total, this work represents an automated mesoscale serial sectioning system for scalable 3D-EM connectomics.

## Introduction

Serial section electron microscopy (ssEM) has proven to be a powerful tool for the investigation of the brain, from analyzing millimeter-scale neuronal circuits to studying localized ultrastructure (Hildebrand et al., 2017; Zheng et al., 2018; Karimi et al., 2019). To this end, the ability to process serial sections, i.e., the cutting of sections on an ultramicrotome and placing the sections onto an EM substrate, has remained a critical and challenging step in ssEM. In recent years, research groups using ssEM have diverged into two primary camps: (1) millimeter-scale ssEM, which uses predominantly automated tools, collects petabyte-sized datasets composed of 10^4^–10^5^ serial sections, and investigates questions of neuronal circuit connectivity (Hayworth et al., 2014; Kasthuri et al., 2015; Lee et al., 2016; Hildebrand et al., 2017; Zheng et al., 2018; Karimi et al., 2019); (2) “traditional” ssEM, which employs predominantly manual techniques, collects gigabyte-sized datasets composed of 10^0^–10^1^ sections, and investigates questions of localized neuroanatomy (de Lima et al., 2012; Kuwajima et al., 2013; Androuin et al., 2018; Burgoyne et al., 2018; Hafner et al., 2018). As a result, serial sectioning has become more specialized and is predominantly conducted, correspondingly, one of two ways: automated tape ultramicrotome (ATUM) serial sectioning for millimeter-scale ssEM (Hayworth et al., 2006) and ribbon-based, manual serial sectioning for traditional ssEM (Harris et al., 2006). While the field has investigated a wide range of neurobiological questions, from dendritic spine geometry to large-scale cortical wiring diagrams, there remains an important need to collect and study mesoscale datasets, i.e., terabyte-sized datasets composed of ∼10^2^–10^3^ serial sections (Bock et al., 2011; Lee et al., 2016; Zheng et al., 2018; Chirillo et al., 2019). Within the mesoscale domain, a variety of neurobiological questions remain to be answered, e.g., questions of synaptic vesicle density, axonal fasciculation patterns, and neuronal ultrastructural variation. In each of these examples, a few dozen serial sections would be insufficient to answer these questions, while several thousand serial sections would be impractical. Thus, there is a need to develop appropriate tools for mesoscale serial sectioning to enable broader access to ssEM and accelerate the pace of neurobiological investigation.

The first described methods for collecting serial sections originate from the mid-1950s, where ribbons of sections are manually picked up onto a slot grid for transmission EM (TEM; Dowell, 1959; Ware and LoPresti, 1975; Harris et al., 2006). This methodology has remained a standard in the field of ssEM, due to its convenience and relative ease when processing a few dozen serial sections. While a variety of methods to reduce or completely remove human skill from serial sectioning have been described (Barnes and Chambers, 1961; Westfall and Healy, 1962; Lee et al., 2018), the automation of serial has converged on continuous, tape-based approaches (Hayworth et al., 2006). While this method is efficacious for collecting thousands of serial sections, the subsequent imaging of the sections is non-trivial. Conventional scanning EM (SEM) is prohibitively slow (Briggman and Bock, 2012), while TEM requires customization of a TEM for tape substrate imaging (Bock et al., 2011; Zheng et al., 2018). (As an aside, advances in multibeam SEM have enabled high-throughput SEM imaging, the limitation now being the cost of a multibeam SEM; Eberle et al., 2015.) For whichever imaging modality, storing, accessing, and analyzing datasets that are composed of thousands of images, i.e., several petabytes of data, is not a trivial task. Thus, there remains a need for an automated serial sectioning methodology specifically designed for mesoscale ssEM studies.

In the following, we describe an automated, mesoscale serial sectioning platform that uses a curvature-induced capillary-interaction-based (i.e., capillary-based), and hydrodynamic force-based (i.e., Stokes-based) trap to passively constrain sections with high accuracy and repeatability. In this method, individual sections are constrained in a stable force-equilibrium state akin to that of kinematic couplings used in precision machine design (Slocum, 1992; Rothenhöfer et al., 2013; Lee et al., 2018a). Subsequently, the sections are picked-up with a loop end-effector that is rigidly affixed to a robotic, three-axis precision linear stage system. The end-effector is calibrated such that its pickup location is matched to a predetermined trapping location; as a result, serial sections are collected without the need for feedback control. Once removed from the waterboat, sections are placed directly onto a heated electron or light microscopy substrate for downstream imaging. In total, we design, fabricate, and characterize, with mathematical modeling and experimental validation, an open-loop, automated mesoscale serial sectioning system for scalable 3D-EM connectomics.

## Materials and Methods

**Figure 1.**
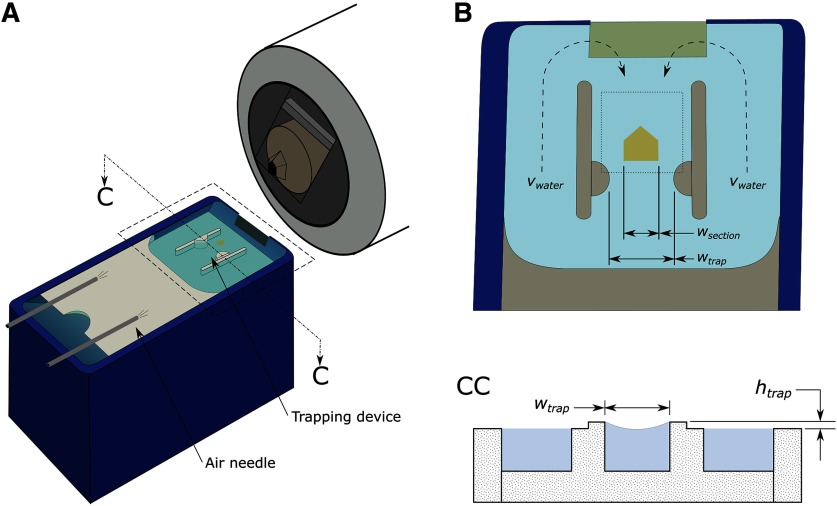
Diagram of diamond knife waterboat with trapping device installed. ***A***, The trapping device, shown within the waterboat, is composed of two semicircular trapping posts and two parallel walls that separate the waterboat into three channels. When the water level is set to a typical cutting level, the channel walls do not protrude significantly from the water; the trapping posts, on the other hand, protrude roughly 1 mm from the nominal water surface, thereby creating curvature-induced capillary interactions (see cross-section view ***CC***). Air needles are attached to the distal end of the waterboat to provide hydrodynamic forces. ***B***, Top view of trapping device, corresponding to the region bounded by the dashed line in ***A***. The air needles supply pressurized air which induce a symmetric water flow pattern with average water velocity, *v_water_*, as shown. The forces trapping the section are modulated by the section size, *w_section_*, the trap width, *w_trap_*, the trap height, *h_trap_* (see cross-section view ***CC***), and the average water velocity, *v_water_*. ***CC***, Cross-sectional view of the trapping device at the trapping posts. Outside of the center channel, the water level remains flat, as shown. Near the trapping posts, the water is pins to the height of the trapping posts, *h_trap_*, thereby creating local curvature in the water surface.

In this system, individual sections are cut using a diamond knife on a conventional ultramicrotome into an adjoining waterboat. Prior to sectioning, our trapping device is installed within the waterboat, as shown in Figure 1*A*, otherwise, the ultramicrotome setup is unchanged from conventional ultramicrotome setup.

The section is transported away from the knife edge after it is cut and towards the trap via water flow, i.e., via hydrodynamic forces, as illustrated in Figure 1*B*. As a secondary measure, to overcome adhesion forces between the section and knife where hydrodynamic forces were not sufficient, an eyelash end effector, that was rigidly affixed to the robotic linear stage system, was used to automatically move the section away from the knife edge. Upon reaching the trap, sections are repelled by curvature-induced capillary forces, establishing a static equilibrium. From this location, the section is picked up by a loop end-effector and placed onto a heated imaging substrate, e.g., silicon wafer, glass slide, in a prespecified pattern such that the order of the serial sections is known. In the loop end-effector, the section is held in place via surface tension forces; on placement on the heated substrate, the residual water evaporates and the section lies down onto the substrate in a wrinkle-free fashion. The loop end-effector, being rigidly affixed to a robotic linear stage system, is calibrated to match the trapping location of the section; thus, sections are collected in an open-loop fashion, i.e., without the need for feedback control.

### Experimental methods

**Figure 2.**
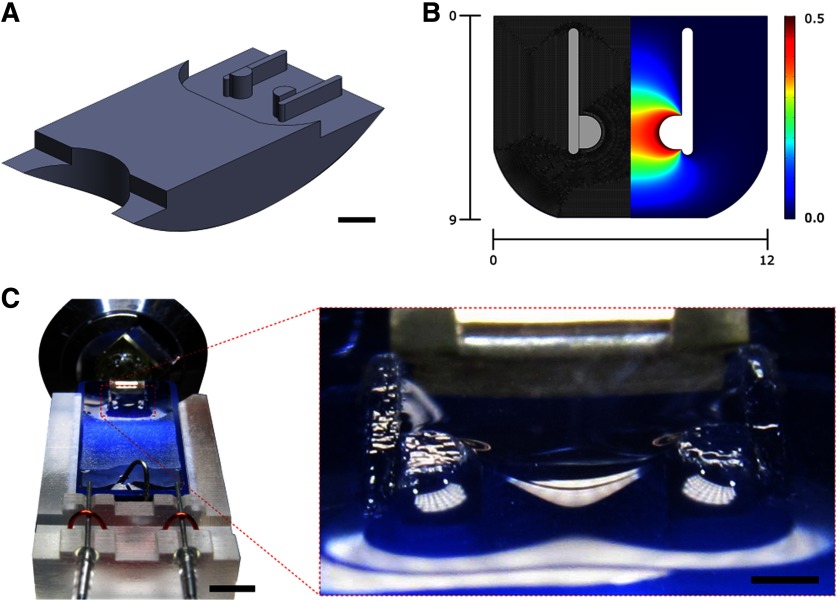
CAD model with finite element analysis. ***A***, Isometric view of the trapping device designed in SolidWorks. This model has a trap width of 3.0 mm and a trap height of 0.5 mm. Scale bar: 3 mm. ***B***, Top view of Young–Laplace equation solution domain. The domain is split symmetrically along the centerline, with the left side showing the finite element mesh and the right side showing the finite element solution for the interfacial height. ***C***, Photograph of experimental setup with inset showing the induced curvature between the trapping posts. The trapping device is shown installed in the waterboat with water filled to appropriate height for sectioning. Air needles are mounted on the distal end of the waterboat using a custom fixture, which provide the hydrodynamic forces. A metal tube is shown protruding from the distal end of the waterboat used for modulating water level. Scale bar: 5 mm.

Bulk resin blocks (EPON812) were trimmed manually to the appropriate cross-sectional area (∼1.5 × 1.5 mm). Resin blocks were placed within the ultramicrotome (Leica UC7) sample chuck and not removed until all experiments trials were completed. Section trapping devices were designed using computer-aided design (CAD) software (SolidWorks). An example of a trapping device is shown in Figure 2*A* with corresponding finite element model mesh grid and solution in Figure 2*B*.

All devices were designed to interface with a Diatome Ultra 45 diamond knife with standard waterboat. Subsequently, design files were postprocessed for 3D printing using CAM software (PreForm) and fabricated using a stereolithography (SLA) 3D printer (Formlabs, Form 2, Clear Resin). Upon completion, device dimensions were manually verified for accuracy.

Prior to sectioning, the trapping device was placed within the diamond knife waterboat (Fig. 2*C*). Being designed for the waterboat, the trapping device sits level with the outer walls of the waterboat (Fig. 2*C*). Once installed, the waterboat is filled with water, as typically conducted for ultramictromy, and aligned with the resin block. Two needles were placed symmetrically at the distal end of the waterboat to induce symmetric water flow patterns within the waterboat (Fig. 2*C*). The needles were mounted to the waterboat using a custom fixture and were connected to a pressurized air cylinder. Air flow was regulated using a precision pressure regulator (Omega/ProportionAir QPV Series). A calibration was conducted to correlate the pressure regulator control voltage with water velocity.

For characterizing and testing the section trapping device accuracy and repeatability, two experiment paradigms were used: single section testing and multisection testing. For single section testing, the same section was trapped 10 times across 10 trials. In each trial, a video of the section was recorded for at least 10 s at ∼10 fps. For multisection testing, 10 different sections were trapped once, thus composing 10 trials. Between trials, the previous section was removed from the waterboat and discarded and a new section was cut. In each trial, a video of the section was recorded for at least 10 s at 10 fps. In all experiments, the water level was maintained manually, using a syringe, to minimize effects due to water evaporation. For each trap design paradigm, 10 single section trials, i.e., trapping of the same section 10 times, and 10 multisection trials, i.e., 10 unique sections each trapped once, were conducted and videos of each trial were recorded. These videos were analyzed *post hoc* to extract the section centroids and quantify the trap accuracy and repeatability. Videos were imported into MATLAB for accuracy and repeatability analysis. For each video, a custom script was used to automatically identify the section centroid in each frame. For videos where contrast was insufficient for automated centroid identification, manual region of interest (ROI) selection was used. For accuracy measurements, the centroid measurements were compared with a fixed origin or “target” defined as the midpoint between the trapping posts and along the waterboat centerline, as shown in Figure 1*B*. For repeatability measurements, we used the SD of the centroid measurements.

In designing our device for trapping serial sections, we performed a parameterization study to understand the effect of various trap parameters. We fabricated and tested eight different device designs: four designs to vary the trap width (*w_trap_* = 1.5–3.0 mm) while holding the trap height constant (*h_trap_* = 0.5 mm) and four designs varying the trap height (*h_trap_* = 0.5–0.84 mm) while holding the trap width constant (*w_trap_* = 3.0 mm), as defined in Figure 1*B*,*C*. For all trap width modulation experiments, sections were cut at nominally 250 nm; for all trap height modulation experiments, sections were cut at nominally 200 nm. The system was calibrated such that average water velocity was ∼1 mm/s to ensure laminar flow. From these experiments, an optimal trap design was selected and long-term automated serial sectioning experiments were conducted; for long-term automated serial sectioning experiments, sections were cut at 250 nm. The cutting speed for the ultramicrotome was set to 0.30 mm/s for all experiments.

During long-term serial sectioning experiments, an individual section is cut using a diamond knife (Diatome) on a conventional ultramicrotome (Leica UC7) into an adjoining waterboat. In cases where the section stuck to the knife edge, an eyelash end-effector, rigidly affixed to a robotic linear stage system (ThorLabs) and manually calibrated such that the eyelash end-effector would detach sections stuck to the knife edge, was used to remove the section from the knife edge. Upon being transported to the trap, the section is picked up by a loop end-effector (TedPella, inner diameter = 2.5 mm) and placed onto an adjacent heated imaging substrate (∼95°C), e.g., silicon wafer, glass slide, in prespecified grid pattern. The loop end-effector is rigidly affixed to the robotic linear stage system and is manually calibrated to match the trapping location of the section. Once all the water has evaporated from the loop end-effector and the section has dried down onto the substrate, the loop end-effector returns to the pickup location, but above the water level, to await the next section for pickup. Sections were placed in a 14 × 9 grid with 3-mm spacing, equating to 126 sections per trial. Trials were limited to this number due to the size of the substrate. Between trials, the substrate was replaced with a new, empty substrate, the diamond knife was cleaned, and the loop and eyelash end-effectors were re-calibrated. During these experiments, a syringe pump (Harvard Apparatus) was used to maintain a constant water level: 6.5 μl of water was added with each section that was cut and removed from the waterboat. Adjustments to the water level were made manually roughly every 20 sections. The ultramicrotome was placed within an enclosure to isolate the system from macroscopic temperature or humidity changes as well as shield the ultramicrotome from external disturbances, e.g., air currents and vibrations.

Samples were prepared for EM using previously published methods for EM staining (Hua et al., 2015). Prior to SEM imaging, samples were imaged on a Zeiss Smartzoom 5 automated digital microscope to locate fiducial markers and obtain low-magnification mosaic images. SEM imaging was conducted using a multibeam SEM (Zeiss MultiSEM 506) at 30 kV. TEM was conducted using a JEOL 1200EX-II with accelerating voltage 120 kV. Samples placed onto glass slides for light microscopy were stained with toluidine blue for 30 s at 80°C and then imaged using a Leica DM6 microscope.

### Mathematical modeling

A mathematical model was used to predict the trapping location of each trap design. The model is composed of two force contributors leading to a static equilibrium: curvature-induced capillary interactions and hydrodynamic forces. To model the effect of curvature-induced capillary interactions, we first needed to solve the Young–Laplace equation for each of our trap designs. In general, the Young–Laplace equation relates the shape of a fluid-fluid interface (i.e., water-air interface) to the difference in capillary pressure. By solving this equation, we are able to obtain the height of the water (*h*) at all points within the trap. Subsequently, we use the solution for height of the water to calculate the water height Laplacian (∇^2^*h*), which we then use to compute the capillary force acting on a section within our trap. To model the effect of hydrodynamic forces, we used the Stokes’ drag force formulation. All parameters within our model were matched to that of experimental parameters, e.g., mean water velocity, section thickness. Using a force balance, we created a mathematical model to predict the centroid trapping location of a section for each trap design.

#### Curvature-induced capillary interactions

For each trap design, the model file was imported into a finite element analysis software (COMSOL) to solve the Young–Laplace equation for the water height within the device domain, as shown in Figure 2*B*. A two-dimensional domain matching the waterboat wetting conditions was selected, and a mesh was automatically generated, limiting the maximum element size to 0.025 mm to ensure sufficient spatial resolution of the solution and solution convergence, as shown in Figure 2*C*, left. In setting the boundary conditions, the water height at the trapping posts was set to match the height of the trapping posts, ranging from *h *=* *0–0.84 mm, while all other boundaries were set to a water height of zero. Upon solving the Young–Laplace equation, written as

(1) Δp=2γH,

where Δ*p* is the Laplace pressure for the water-air interface, *γ* is the surface tension coefficient for a water-air interface at 25°C, and *H* is the mean curvature of the fluid-fluid interface, the solution for the surface height (*h*) and surface Laplacian (∇^2^*h*) were exported as text files; an example of the surface height solution is shown in Figure 2*B*, right. These solutions were then imported into a custom MATLAB script to calculate the capillary force at each point in the domain. From prior literature, the curvature-induced capillary force, *F_c_*, can be written as

(2) $$F_{c}=2\pi \gamma H_{p}R_{p}^{2}\nabla^{2}h,$$

where *γ* is the surface tension coefficient for a water-air interface at 25°C, *H_p_* is the mean water deformation amplitude surrounding the section, *R_p_* is the particle radius, i.e., the section thickness, and $\nabla^{2}$ *h* is the surface height Laplacian (Stamou et al., 2000; Cavallaro et al., 2011).

#### Hydrodynamic force modeling

The Stokes’ law drag formula for thin sheets can be written as

(3) $$F_{d}=4\pi \mu L_{c}C_{d}v,$$

where *μ* is the viscosity of water at 25°C, *C_d_* is the drag coefficient of a thin plate, *v* is the average water velocity, taken from our calibration curve, and *L_c_* is the characteristic length of the section, defined as

(4) $$L_{c}=\sqrt{w_{section}^{2}+h_{section}^{2}+t^{2}},$$

where *w_section_*, *h_section_*, and *t* are the section width, height, and thickness, respectively (Saffman, 1976; Stamou et al., 2000; Cavallaro et al., 2011). (Derivations of Equations 2, 3 are provided below, Derivation of Stokes’ law.) Given the density and viscosity of water at 25°C, the size of the trapping device (∼1 mm), and the average water velocity (∼1 mm/s), we calculate a Reynolds number of order unity, thus we assume laminar flow. The average water velocity was measured only for the trapping domain, thus, we assume the Stokes’ drag force calculation to be valid within the trapping domain. By subtracting the calculated Stokes’ drag force, *F_d_*, from the curvature-induced capillary force, *F_c_*, and looking for the location where these two forces are equal and opposite in magnitude and direction, respectively, we are able to predict the trapping location of a section within the trapping device.

#### Derivation of Stokes’ law

The derivation of the formula describing the drag force acting on a sphere of radius a and velocity U, moving through a viscous fluid with density ρ and viscosity μ, was first described by G. Stokes in 1851. In the following, we re-derive Stokes’ law and give further commentary on its applicability to thin sheets, i.e., ultrathin sections, moving through a fluid.

The Navier–Stokes equations for an incompressible Newtonian fluid can be written as

(3-1) $$0=-\nabla p+\mu \nabla^{2}u$$

(3-2) 0=∇⋅u,

where Equation 3-1 represents the conservation of momentum, while Equation 3-2 represents the conservation of mass. Recalling the vector calculus identity

$$\nabla \left(\nabla \cdot u\right)-\nabla^{2}u=\nabla \times (\nabla \times u),$$

and applying Equation 3-2, we are able to rewrite Equation 3-1 as

(3-3) ∇p=−μ(∇×(∇×u))

Next, we introduce a spherical coordinate system with a sphere of radius r=a and origin placed at the sphere’s center. Furthermore, we assume axisymmetric flow so that u is independent of φ in our spherical coordinate system. This assumption, combined with the previous assumptions of incompressible flow in a Newtonian fluid, allows us to relate the flow velocity vector, u, with the Stokes stream function, ψ, written in component form as

(3-4) $$u_{r}=\frac{1}{r^{2}sin\theta }\frac{\delta \psi }{\delta \theta }$$

(3-5) $$u_{\theta }=-\frac{1}{rsin\theta }\frac{\delta \psi }{\delta r}.$$

As an aside, stream functions are useful in that they allow us to solve the incompressible Navier-Stokes equation by imposing a relationship between the velocity components and partial derivatives of the stream function. Equations 3-4, 3-5, given above, come naturally from Equation 3-2, given axisymmetric flow.

Recalling vorticity, ω, defined as

(3-6) ω≡∇×u,

and applying Equations 3-4, 3-5, we can see the vorticity vector is equal to

(3-7) $$\omega =[\begin{array}{c} 0\\ 0\\ -\frac{1}{rsin\theta }\left(\frac{\delta^{2}\psi }{\delta r^{2}}+\frac{sin\theta }{r^{2}}\frac{\delta }{\delta \theta }\left(\frac{1}{sin\theta }\frac{\delta \psi }{\delta \theta }\right)\right) \end{array}],$$

where the only non-zero component is the azimuthal component, as expected due to the assumption of axisymmetric flow. Equation 3-7 can be written more succinctly as

(3-8) $$\omega_{\phi }=-\frac{1}{rsin\theta }L\mathrm{\psi ,}$$

where *L* is a differential operator defined as

(3-9) $$L\equiv \frac{\delta^{2}}{\delta r^{2}}+\frac{sin\theta }{r^{2}}\frac{\delta }{\delta \theta }(\frac{1}{sin\theta }\frac{\delta }{\delta \theta }).$$

Applying Equations 3-8, 3-9 to Equation 3-3, we can rewrite the conservation of momentum as

(3-10) $$\nabla p=-\mu \nabla \times \omega =[\begin{array}{c} \frac{-\mu }{rsin\theta }\left(\frac{\delta }{\delta \theta }(\omega_{\phi }sin\theta \right)\\ -\frac{\mu }{r}\left(\frac{\delta }{\delta r}\left(r\omega_{\phi }\right)\right)\\ 0 \end{array}].$$

Furthermore, recalling the vector calculus identity,

(3-11) ∇⋅(∇×A)=0,

we can rewrite the conservation of momentum equation as

(3-12) $$\nabla \cdot \nabla p=-\mu \nabla \cdot \left(\nabla \times \omega \right)=\nabla \cdot \left[\begin{array}{c} \frac{-\mu }{rsin\theta }\left(\frac{\delta }{\delta \theta }(\omega_{\phi }sin\theta \right)\\ -\frac{\mu }{r}\left(\frac{\delta }{\delta r}\left(r\omega_{\phi }\right)\right)\\ 0 \end{array}\right]=0.$$

Applying Equations 3-8, 3-9, we arrive at

(3-13) $$\frac{1}{r^{2}}\frac{\delta }{\delta \theta }\left(\frac{1}{sin\theta }\frac{\delta }{\delta \theta }\left(L\psi \right)\right)+\frac{\delta }{\delta r}\left(\frac{1}{sin\theta }\frac{\delta }{\delta r}(L\psi )\right)=0.$$

To solve this partial differential equation, we apply separation of variables in the form

(3-14) $$\psi =\sin^{2}\theta f(r)$$

to Equation 3-13 and obtain

(3-15) $${\left(\frac{{\text{δ}}^{2}}{\delta r^{2}}-\frac{2}{r^{2}}\right)}^{2}f=0.$$

We assume f(r) to take the form

(3-16) $$f\left(r\right)=r^{\lambda }.$$

Substituting this into Equation 3-15, we obtain

(3-17) $$\left({\text{λ}}^{2}-1\right)\left(\text{λ}-2\right)\left(\text{λ}-4\right)=0$$

Thus, the general solution of Equation 3-13 is

(3-18) $$f\left(r\right)=\frac{A}{r}+Br+Cr^{2}+Dr^{4}.$$

Applying the boundary conditions

(3-19) $$u_{r}=u_{\theta }=0{|}_{r=a},$$

(this can be thought of as the “no-slip” condition on the surface of the sphere) and

(3-20) $$\psi =\frac{1}{2}r^{2}sin^{2}\theta U{|}_{r\to \infty },$$

i.e., the velocity approaches the free-stream velocity far from the sphere, we write the values for the coefficients A, B, C, and D to be

$$A=\frac{1}{4}Ua^{2},B=-\frac{3}{4}Ua,C=\frac{U}{2},D=0.$$

Thus,

(3-21) $$\psi (r,\theta )=\frac{1}{4}U\left(\frac{a^{3}}{r}-3ar+2r^{2}\right)\sin^{2}\theta .$$

Furthermore, recalling Equations 3-4, 3-5, 3-10, we can write

(3-22) $$u_{r}(r,\theta )=U\left(\frac{a^{3}}{2r^{3}}-\frac{3a}{2r}+1\right)cos\theta ,$$

(3-23) $$u_{\theta }(r,\theta =U\left(\frac{a^{3}}{4r^{3}}+\frac{3a}{4r}-1\right)sin\theta ,$$

(3-24) $$p(r,\theta )=\left(\frac{3a\mu U}{2r^{2}}\right)cos\theta .$$

To calculate the drag force acting on the sphere, we can sum the forces due to the pressure, i.e., forces normal to the sphere surface, and forces due to the viscous shear, i.e., forces tangent to the sphere surface. These can be calculated as

(3-25) $$F_{pressure}=2\pi a^{2}\overset{\pi }{\underset{0}{\int }}p\left(r,\theta \right)sin\theta cos\theta d\theta =2\pi a\mu U$$

and

(3-26) $$F_{shear}=2\pi a^{2}\overset{\pi }{\underset{0}{\int }}\tau_{r\theta }\sin^{2}\theta d\theta =4\pi a\mu U,$$

where $\tau_{r\theta }$ is defined as

(3-27) $$\tau_{r\theta }=-\mu \left(r\frac{\delta }{\delta r}\left(\frac{u_{\theta }}{r}\right)+\frac{1}{r}\left(\frac{\delta }{\delta \theta }u_{r}\right)\right)$$

Thus,

(3-28) $$F_{drag}=F_{pressure}+F_{shear}=6\pi a\mu U.$$

Hence, we are left with Stokes’ law, as written in Equation 3-28 (Stokes, 1851). For the case of a cylinder, with radius *a* and length *h*, rotating in a fluid, the drag force acting on the cylinder can be written as

(3-29) $$F_{drag}=4\pi \mu hC_{d}U\left(\text{Saffman},\;\text{1976}\right)$$

where μ is the fluid viscosity, $C_{d}$ is the shape-factor (for consideration of objects with finite size), and *U* is the free-stream velocity. Thus, we take this case to be equivalent to that of an infinitesimally thin sheet (i.e., ultrathin section) moving along interface of two fluids, with one fluid having significantly greater viscosity than the other, i.e., the water-air interface.

## Results

**Figure 3.**
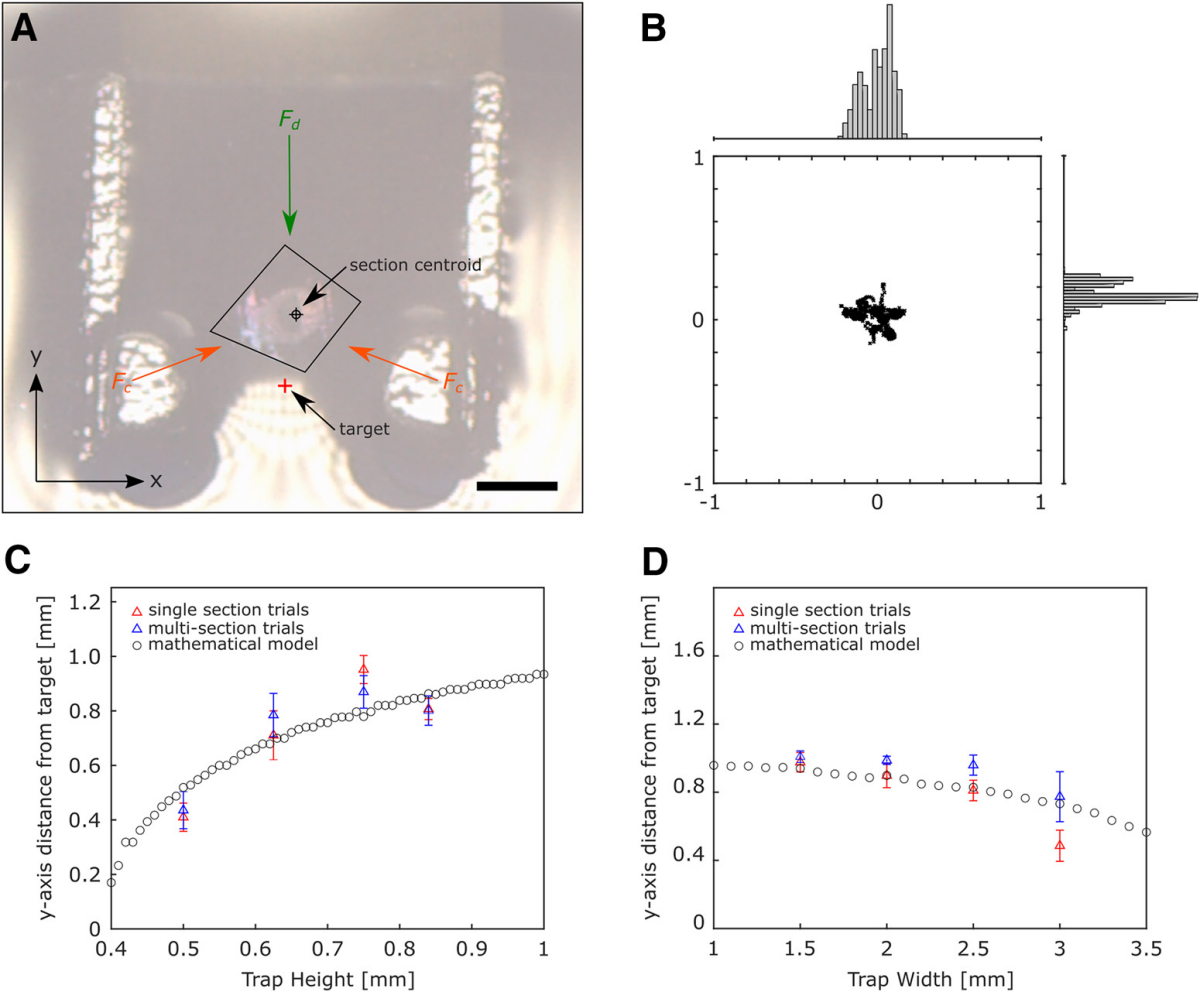
Trap design parameterization experiment and modeling results. ***A***, Single frame showing an individual section trapped within the trapping device. The section is trapped via balance of curvature-induced capillary interactions, *F_c_* (orange), and Stokes drag forces, *F_d_* (green). The calculated centroid (black crosshair) and the defined target (red cross) are shown. The orientation of the *x*- and *y*-axes relative to the trapping device is shown in the bottom left. Scale bar: 1 mm. ***B***, Scatter plot of section centroid positions for a single trap design with w_trap_ = 2.5 mm, h_trap_ = 0.5 mm, w_section_ = 1.5 mm. Ten sections were individually trapped and their positions recorded over time. For each section, we analyzed its local movement within the trap over 10 s; videos were recorded at 10 fps. All of the centroid positions are shown from all 10 trials (black x). The x-component centroid position distribution is shown above the scatter plot (x_st. dev._ = 91 μm); the y-component centroid position distribution is shown to the right of the scatter plot (y_st. dev._ = 62 μm). The centroids are plotted relative to the mean centroid position. Plot axes are given in millimeters. ***C***, Distance between the mean centroid position and target along the *y*-axis plotted versus the trap height. The mathematical model (black circles) shows a non-linear increase in the distance between the mean centroid position and target along the *y*-axis as the trap height increases. This trend shows good alignment with our single section (red) and multisection (blue) experiment results (RMSE = 0.27 mm). ***D***, Distance between the mean centroid position and target along the *y*-axis plotted versus the trap width. The mathematical model (black circles) shows a non-linear decrease in the distance between the mean centroid position and target along the *y*-axis as the trap width increases, showing good alignment with our single section (red) and multisection (blue) experiment results (RMSE = 0.31 mm).

The device as designed and fabricated in shown in Figure 2. An image of an individual section trapped within the device is shown in Figure 3*A*.

For this trial, the trap width was set to 2.5 mm and the trap height to 0.5 mm. From this image, we can see that the section is trapped between and above the semicircular trapping posts due to the balance of curvature-induced capillary interactions (Fig. 3*A*, orange) and Stokes’ drag force (Fig. 3*A*, green).

The section’s centroids over a 10-s duration is shown in Figure 3*B*, plotted with respect to its mean centroid position within this duration. The distance along the *y*-axis between the mean centroid position and the target, defined as the midpoint between the trapping posts and along the waterboat centerline, was calculated and plotted against the pertinent design parameter (trap height or trap width), as shown in Figure 3*C*,*D*, respectively.

**Figure 4.**
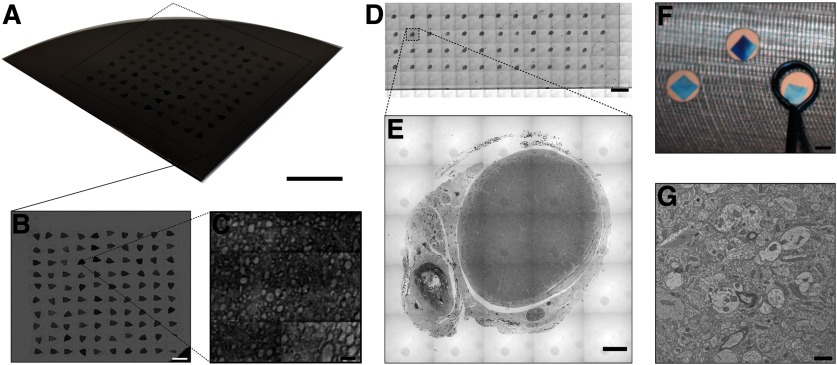
Examples of serial sections placed onto conventional light and EM substrates. ***A***, Photograph of 100 serial sections of mouse brain tissue of nominal thickness 60 nm placed onto a silicon wafer. Scale bar: 10 mm. ***B***, Top-view light micrograph of 100 serial sections placed onto a silicon wafer. Sections are placed in a raster-grid formation, with section 1 being on the bottom left corner, section 2 being above section 1, and section 100 at the top right corner. Scale bar: 3 mm. ***C***, Scanning electron micrograph imaged using a multibeam SEM. Myelinated axons can be observed for potential sparse reconstruction of neuronal networks. Scale bar: 10 μm. ***D***, Mosaic low-magnification light micrograph of 52 rat optic nerve serial sections cut at 250 nm and placed onto a glass slide. Sections are stained with toluidine blue for optical contrast. Scale bar: 3 mm. ***E***, Mosaic high-magnification light micrograph of a rat optic nerve section. Individual axons can be observed within the optic nerve. Scale bar: 100 μm. ***F***, Image of three serial sections (nominal thickness 40 nm) placed onto an aluminum substrate with imaging apertures covered with Luxel support film for TEM. The loop end effector used to pick-up and placed sections is shown. Scale bar: 1 mm. ***G***, Representative high-magnification transmission electron micrograph of an ultrathin human cortical brain tissue section. Scale bar: 1 μm.

Using a trapping device with trap width and height equal to 2.5 and 0.5 mm, respectively, we performed long-term automated serial sectioning experiments, placing the sections onto a variety of substrates to demonstrate the utility of the system. A photograph of a series of 100 serial sections (mouse cortical tissue, nominal section thickness = 60 nm) is shown placed onto a silicon wafer in Figure 4*A*.

A top-view low-magnification light micrograph of the same sections is shown in Figure 4*B*, and a high-magnification scanning electron micrograph is shown in Figure 4*C*. A mosaic, low-magnification light micrograph of 52 serial sections (rat optic nerve tissue, section thickness = 250 nm) is shown in Figure 4*D*. In this experiment, sections were placed onto a glass slide and stained for optical contrast with Toluidine blue. A high-magnification image of an individual optic nerve section is shown in Figure 4*E*, and an image of three serial sections (human cortical tissue, nominal section thickness 40 nm) is shown in Figure 4*F*. These sections are placed on an aluminum substrate with apertures covered with Luxel support film for TEM imaging. A representative high-magnification transmission electron micrograph is shown in Figure 4*G*.

## Discussion

As shown in Figure 3*A*, individual sections are trapped between the two semicircular pillars, i.e., along the channel centerline and lying upstream from the pillars in the positive *y*-axis direction. This is likely facilitated through a balance of curvature-induced capillary interactions and Stokes-based drag forces. In this way, the trap conforms to the exact constraint design principle, which states that the number of points of constraint and number of degrees of freedom (DOF) should be equal (Blanding, 1999). The number of DOF experienced by the section is three: two DOF due to linear translation along and *x*- and *y*-axes, and one DOF due to in-plane rotation. As illustrated in Figure 3*A*, the symmetric semicircular trapping posts provide two capillary-based forces (orange arrows), pointing from the center of the semicircular posts and towards the section centroid, and one restoring, Stokes-based force pointing down (green arrow), i.e., negative y-direction, towards the section centroid. In total, this trapping device represents a non-Hertzian contact-based kinematic coupling that could be useful for trapping soft matter (Young’s modulus, E, ∼1 MPa), as opposed to traditional Hertzian contact-based kinematic couplings used for conventional engineering materials that have Young’s modulus ∼1 GPa and rely on minimal deformation of the trapped material under the influence of the contact and restoring forces (Slocum, 1992; Rothenhöfer et al., 2013).

In analyzing the stability of section trapping over a 10-s duration, as shown in Figure 3*B*, we observe that the distribution of the centroid positions along both the *x*- and *y*-axes remains symmetric about its mean value without an observable bias or skew towards any direction. This is expected as the section has reached a static equilibrium in this trapped configuration, thus we do not expect a bias in the section’s centroid position, which would be caused by an unaccounted external force. While the section is in a stable position, we note that the centroid positions show a non-zero SD. The variability in centroid position within this 10-s duration could be caused by small variations in local water flow, in section orientation, or in water height due to evaporation.

In characterizing the section trapping performance of a function of trap height and width, we found that the distance along the *x*-axis between the mean centroid position and the target to be constant between all trap designs (100 ± 80 μm). This is likely due to the symmetry about the waterboat centerline, i.e., about the *y*-axis as depicted in Figure 3*A*, for all of the trap designs. While this value is constant, we observe a non-zero value although all trap designs share the same symmetry about the waterboat centerline. This could be explained by the asymmetry of the section’s geometry.

Furthermore, in analyzing trends in the mean centroid position along the *y*-axis, we see that for the trap height parameter study, our model (Fig. 3C, black circles) predicts that the distance between the mean centroid position and the target increases as the trap height increases in a non-linear fashion while for the trap width parameter study, our model (Fig. 3*D*, black circles) predicts that the distance between the mean centroid position and the target decreases as the trap width increases in a non-linear fashion. For both the trap height and width parameter studies, the mean centroid positions from our single section (Fig. 3*C*,*D*, red triangles) and multisection (Fig. 3*C*,*D*, blue triangles) experiments shows good alignment our mathematical model without any fitted parameters (RMSE = 0.27 mm, RMSE = 0.31 mm, trap height and width studies, respectively), indicating that the sections are predominantly trapped via a balance of curvature-induced capillary interactions and Stokes-based hydrodynamic forces. From prior literature, it is likely that the curvature-induced capillary interactions are quadrupolar-monopolar in nature (Stamou et al., 2000; Cavallaro et al., 2011; Yao et al., 2015; Lee et al., 2018a). We note that while a trap height of 0.25 mm was tested, this trap height failed to consistently trap sections. Thus, it is likely that a trap height of 0.5 mm forms a functional lower limit for the robust trapping of serial sections. Additionally, we tested a trap height of 1.0 mm; at this trap height, the water fails to pin to the trapping post due to the inability for the water-air interface to assume such an extreme meniscus shape. Hence, a trap height of 0.84 mm represents a functional upper limit for the trapping of serial sections. Arguably, while the trapping of serial sections could still be possible for trap heights >0.84 mm, additional methods would be necessary to know the precise water height at the trapping posts, e.g., interferometry. For the trap width study, we limited our minimum trap width to 1.5 mm as this value approached the section size. Further decrease in trap width would prevent movement of the section due to a physical barrier, i.e., the trapping device would behave as a size filter. Hence, for our section size, a trap width of 1.5 mm represents a functional lower bound for the trapping for serial sections. Additionally, for trap widths >3.0 mm, we did not observe robust trapping of sections; instead, sections flowed freely through the trapping device without observable reduction in velocity as it approached the trapping posts; therefore, a trap width of 3.0 mm, for our section size, represents a functional upper bound for the robust trapping of serial sections.

We see that in both trap design paradigms, the mathematical model capitulates an aliasing or staircase-like effect; this is likely due to the discretization of the domain from the finite element analysis and can likely be reduced by decreasing the mesh element size. While in our analysis, we use absolute values of trap height and trap width, these variables could be non-dimensionalized by relating these values to the section geometry. (As an aside with regards to experiment, for selecting an optimal block face geometry for this technique, we recommend using relatively isometric block face shapes (e.g., a square, rhombus, etc.). We find that using an isometric block face shape produces sections that lie along the trapping channel center line (as opposed to the section position being biased towards one trapping post), which enables ease of section pickup with the loop end effector. A formal study of the block face geometry and its effect on section trapping remains to be conducted.)

Since our system traps sections via a balance of two forces, capillary interactions and Stokes drag forces, this static equilibrium can be equivalently stated as the ratio of these two forces equated to unity. Hence, we introduce a dimensionless quantity, termed the modified capillary number, Ca*, that captures this relationship, written as

(5) $$Ca^{*}=\frac{F_{d}}{F_{c}}=\frac{4\pi \mu L_{c}C_{d}v}{2\pi H_{p}R_{p}^{2}\nabla^{2}h}=\frac{\mu v}{\gamma H_{p}R_{p}\nabla^{2}h}=Ca\frac{1}{H_{p}R_{p}\nabla^{2}h}.$$

Notably, this quantity contains a previously defined dimensionless quantity, the capillary number, *Ca*, as shown in Equation 5. Traditionally, the capillary number describes the ratio of hydrodynamic to surface tension forces. While the device for our system uses a balance of hydrodynamic and surface tension forces, it is important to distinguish the specific type of surface tension forces at play: curvature-induced quadrupolar capillary interactions. This is captured by the terms which modify the traditional capillary number, namely *H_p_*, the deformation amplitude, *R_p_*, the particle size, and ∇*^2^h*, the surface height Laplacian. Moreover, Equation 5 can be used as a functional design constraint. In systems where Ca* ∼ 1, capillary/Stokes-based trapping can be effectively used. For systems where Ca* ≫ 1, drag forces dominate; while for systems where Ca* ≪ 1, capillary interactions have a greater effect.

In selecting an optimum trap design for long-term automated serial sectioning experiments, we analyzed the RMS SEM centroid positions, i.e., the RMS repeatability, combining both the repeatability along the *x*- and *y*-axes into a single value. We found that for the trap height parameter study, a trap height of 0.5 mm ceded the smallest RMS repeatability (60 μm). For the trap width parameter study, while trap widths of 1.5 and 2.0 mm showed the smallest RMS repeatability values (70 μm for both widths), these designs were incompatible with our section pick-up method (loop-based pick-up); therefore, we chose to use a trap width of 2.5 mm, which gave the next-smallest RMS repeatability (100 μm). These values are well below 10% of the section’s characteristic size. By using a loop end-effector to pick-up the sections with an inner diameter of 2.5 mm, which provided sufficient tolerance to accommodate our observed repeatability values, we were able to pick-up sections without a feedback control system.

Upon implementing and using our device to collect serial section datasets, we used SEM (Fig. 4*A–C*), light microscopy (Fig. 4*D*,*E*), and TEM (Fig. 4*F*,*G*) to assess the quality of the tissue after it had been collected using this method. From the photograph of a series of 100 serial sections collected onto a silicon wafer (Fig. 4*A*) as well as the top view image shown in Figure 4*B*, we do not observe any macroscopic defects, e.g., wrinkles or cracks, which may have occurred during the collection process. Because the sections are collected and placed onto a substrate in an automated fashion, the order of the placement of the sections can be prespecified. As in Figure 4*B*, the sections were placed in a raster-grid pattern, i.e., section 1 is located at the bottom left corner with section 2 directly above it and section 100 is located at the top right corner. In Figure 4*C*, a high-resolution scanning electron micrograph is shown, depicting larger myelinated axons and demonstrating the ability for this technique to be used for sparse reconstruction of neuronal networks. With further optimization of tissue staining for SEM, one could enable dense reconstruction of neural tissue as well as the study of subcellular structures using this method. In Figure 4*D*, out of this series of 52 serial sections, we did not observe macroscopic defects, e.g., wrinkles and cracks. This is expected given our ability to collect series of ultrathin sections with high yield, as shown in Figure 4*A*,*B*. While these sections were thicker (nominally 250 nm), we do not expect any fundamental limitations for this method for even thicker sections. It is conceivable that this method would be amenable to handling micron-thickness sections. Furthermore, in Figure 4*E*, at roughly 100× optical magnification, we do not see any defects that may have occurred during our serial section collection process. Additionally, within Figure 4*E*, individual axons are observable within the optic nerve, demonstrating the utility of this method for conventional light microscopy investigation of serial sections. This method could be useful for studying tissue volumes where high in-plane resolution is necessary while high out-of-plane resolution is unneeded. An example of this could be in studying structural variation in the optic nerve and the surrounding tissue along the length of the optic nerve and its relevance to myopathy or other vision degenerative diseases (Li et al., 2019). In demonstrating our system’s compatibility with multiple imaging modalities, Figures 4*F* depicts placement of the sections onto TEM substrates. While these sections were placed onto a custom aluminum TEM substrate coated with a plastic support film (Luxel), it is likely that sections could be placed onto traditional TEM grids with this technique by placing the sections onto grids which lie on a grid coating plate, e.g., PELCO Grid Coating Plate, TedPella. We note that by removing the human user from this conventionally tedious and highly dexterous task, the risk of breaking the support film on the TEM substrate is greatly reduced. The robotically-controlled, loop end-effector is shown placing one section onto the substrate, demonstrating accuracy and consistency in placing the sections on the TEM substrate. Within the high-resolution transmission electron micrograph in Figure 4*G*, cross-sections of axons, dendrites, synapses, and subcellular structures can be observed. While TEM imaging conventionally allows for high in-plane resolution, the drawback, often times, is in the need to collect hundreds of serial sections to a substrate prior to imaging. Thus, our device could be particularly useful for those already performing TEM imaging, which automates this bottleneck.

In total, we demonstrate that this system is amenable to SEM and TEM as well as conventional light microscopy. Additionally, we demonstrated the ability of this system to conduct mesoscale serial sectioning experiments; we collected eight serial section datasets each composed of 126 serial sections with an average section loss rate 0.50% and average throughput of 63 s/section. We see that due to the repeatability of the trapping device and the tolerance afforded by the size of the loop, we are able to repeatably collect ∼10^2^ without section damage, providing a veritable mesoscale serial sectioning method for 3D-EM connectomics. This method can be scaled to larger volumes of tissue by collecting serial sections in a batch-wise process, as previously done (Lee et al., 2018b).

The only failure mode we experienced during our long-term serial sectioning experiments were sections that stuck to the knife edge and as a result, were damaged during collection process. From prior literature, the adherence of sections to the knife edge has been address via a variety of methods, e.g., dissipation of electrostatic charging to prevent stick and physical dislodging of sections via pneumatic actuation (Kolotuev et al., 2012; Lee et al., 2018b). During our experiments, the humidity and temperature was recorded to be between 41–42% and 21–22°C; the water level was controlled within ±5 μl. Cutting and trapping parameters were kept constant between all experiments. The same tissue block was used for all experiments. Thus, with similar experimental parameters, serial sectioning experiments composed of ∼10^2^ can expect <1% section loss rate. For longer serial sectioning experiments (i.e., >10^3^ serial sections), further precision in the control of the experimental parameters is likely necessary if a 1% section loss rate is necessary, when using this serial sectioning method. For larger datasets, a viable alternative may be the implementation of an ATUM-based serial sectioning system. While ATUM-based serial sectioning is capable of collecting mesoscale datasets, certain biological methods require imaging substrates other than Kapton tape, e.g., immunolabeling (Micheva and Smith, 2007; Lam et al., 2015; Fang et al., 2018). Additionally, while ribbon-based serial sectioning is capable of collecting mesoscale datasets, these are typically herculean efforts not readily replicated across research institutions. For many neurobiology labs already equipped with an ultramicrotome and an electron or light microscope, this system could be readily adopted in a piece-meal fashion, e.g., the trapping system could be used without a robotic pick-up system. In this case, the need for highly-trained, dexterous users would be ameliorated, and the traditional serial sectioning workflow would remain mostly unchanged. Another case could be adopting this system for compatibility with a tape-based substrate. As previously mentioned, immunolabeling could provide additional orthogonal information to a serial section dataset; thus by using a robotic pick-and-place system to place sections onto a variety of substrates, e.g., one out of every 10 sections is placed onto a glass slide and processed for immunolabeling while the rest are imaged with EM, one could obtain both neuroanatomical and proteomic data (Randel et al., 2015; Williams et al., 2017).

With recent advances in automated segmentation of serial section EM datasets, the analysis of mesoscale datasets consisting of 10^2^–10^4^ sections becomes possible without significant effort from human annotators. Additionally, due to the amenability of this serial section collection methodology to various substrates, this method could be extended to other substrates, such as silicon nitride for transmission SEM studies (Kuwajima et al., 2013, Lee et al., 2018b). Future work for this methodology may investigate a variety of parameters, such as section thickness, surface tension coefficient, and fluid viscosity. With regards to the study of section thickness, from preliminary results, it appears that the system remains stable within the 50- to 500-nm thickness range. Additionally, this technique could be useful in a broader context of biological sciences. Historically, EM has been a powerful tool for studying cellular ultrastructure; accordingly, the widespread adoption of 3D-EM could lead to new discoveries not only in neuroscience, but also in in molecular and cell biology.

## Conclusion

In total, this work represents an automated mesoscale serial sectioning system for scalable 3D-EM connectomics. From our experiments, we demonstrate the ability to repeatably collect ssEM datasets, composed of 126 serial sections, in an automated fashion with an average loss rate and throughput of 0.50% and 63 s/section, respectively (*n* = 8 trials). Furthermore, we show with light and EM imaging, the ability to collect serial sections onto a variety of electron and light microscopy substrates without significant defects or loss. As shown with modeling and experiment, our trapping device, accurately and repeatability positions sections through a balance of curvature-induced capillary interactions and Stokes-based drag forces. We designed, fabricated, and characterized the trapping device, identifying an optimal design from a parametrization study (RMS repeatability = 100 μm), thereby enabling collection of sections using open-loop control. Computationally, our mathematical model accurately predicts the trapping position of the sections over a range of trapping parameters (RMSE = 0.27 mm). Experimentally, our device interfaces with a conventional ultramicrotomy diamond knife, accomplishing in-line, exact-constraint trapping of sections within the waterboat. This design, model, and experiment extends the modeling of water-air interface forces as well as demonstrates a useful tool for mesoscale serial sectioning EM, an important need in the field of neuroanatomy, connectomics, and neuroscience.

## Synthesis

Reviewing Editor: Leonard Maler, University of Ottawa Decisions are customarily a result of the Reviewing Editor and the peer reviewers coming together and discussing their recommendations until a consensus is reached. When revisions are invited, a fact-based synthesis statement explaining their decision and outlining what is needed to prepare a revision will be listed below. The following reviewer(s) agreed to reveal their identity: Uri Manor.

Editorial review and comments

This manuscript gives a coherent and practical method for collecting 100s of ultrathin sections in serial order. In addition, it provides detailed computational justification for all the critical steps of the method.

The actual technical descriptions of the methods were clearly written and any lab does EM on a regular basis should be able to adopt this procedure without difficulty. The suggestion by the authors on how to adopt the method is stages was especially practical. The only difficulties I encountered in the manuscript were in the Mathematical Modeling section. I am familiar with applications of dynamical systems theory and stochastic methods for neuroscience. However, my understanding of fluid mechanics is negligible and I had a very hard time (and checking of textbooks) following the detailed arguments in this section. I suspect that few EM labs have such knowledge. Fortunately, I do not think that this section is vital to the practical use of the methods described. It would, for the sake of the few EM practitioners with a deeper background, to have a more extensive explanation of the authors’ analyses. These could be placed in Supplementary Materials and so hidden from the majority of end users. I list the pertinent points below.

1) Stokes drag formulation (I think this is derived from the Navier-Stokes equations but clarification would be nice). The equation (#3) is given but an expanded version that gives soe intuitive insight would be nice - I, at least, would have enjoyed this.

2) Surface Laplacian. I did remember the Laplacian but not sure what comes out when applied to the Young-Laplace equation.

I fully agree with all the points noted by the reviewer and am also eager to see this paper published and the methods presented widely adopted.

Reviewer 1

This paper provides a theoretically grounded method for designing a hydrodynamic trap for an ultrathin section, using a force equilibrium of water flowing in a precisely modeling chamber to keep the section from moving less than a millimeter, which is then be targeted for automatic pickup and deposition on a substrate. The process, taking just over a minute can be repeated with very low failure rate (up to 126 sections). It is low-cost (3d printable parts for the trap, linear stage and linear actuator for the loop) and effective technique that will be immediately useful to many cell biologists interested in volumetric properties of their tissues. So this is an advance not just for neuroscience but really for any area of biology that benefits from 3DEM analysis.

This paper is a wonderful blend of modeling, design, and a very practical application that has been overlooked - namely, serial sectioning for SEM on a silicon substrate. By collecting one section at a time, the risk of out of order ribbons is eliminated - for this conceptual contribution alone the paper is worthy of publication (although it’s been reported before, but for pickup on silicon nitride windows which is more esoteric and less applicable).

This paper is important. It provides a theoretically grounded method for designing a hydrodynamic trap for an ultrathin section, using a force equilibrium of water flowing in a precisely modeling chamber to keep the section from moving less than a millimeter, which is then be targeted for automatic pickup and deposition on a substrate. The process, taking just over a minute can be repeated with very low failure rate (up to 126 sections). It is low-cost (3d printable parts for the trap, linear stage and linear actuator for the loop) and effective technique that will be immediately useful to many cell biologists interested in volumetric properties of their tissues. The authors may want to think about mentioning how this technique has applicability beyond just neuroscience/connectomics.

Without the trapping device, fluid force modeling, and robotic deposition, this would still be a big conceptual contribution that will be immediately useful to many labs that have access to an ultramicrotome and SEM. A logical and important extension of LASSO: using silicon chips and an SEM instead of custom silicon nitride window arrays and a custom TEM/tSEM sample holder.

I want to see at least 5 300+ section series’ and their respective loss rates. I want to see at least 1 600 section series and a loss rate. At 1 minute per section, shouldn’t be too time consuming to generate.

One question we have is: What happens when section/block face shape “changes”? What is the optimal block face shape for this technique?

“While in our analysis, we use absolute 370 values of trap height and trap width, these variables could be non-dimensionalized by relating these 371 values to the section geometry.” We would like to know more about choosing block face geometry.

One more question we have is: How did you use your loop end effector to deposit sections on slot grids?

We hope these relatively minor suggestions are quick enough to address with a very fast revision turnaround - this paper deserves to see the light of day sooner rather than later.
